# Contributing factors of delay in seeking treatment for childhood diarrheal diseases in Berbere Woreda, Ethiopia: an unmatched case–control study

**DOI:** 10.1186/s41043-023-00411-4

**Published:** 2023-07-10

**Authors:** Mebratu Bekele, Megersso Urgessa, Kebede Kumsa, Edao Sinba

**Affiliations:** 1 Negelle Health Center, Negelle Town, West Arsi Zone, Ethiopia; 2 Department of Public Health, School of Health Science, Shashemene Campus, Madda Walabu University, P.O.BOX: 238, Shashemene, Ethiopia

**Keywords:** Delayed care seeking, Caregivers, Childhood diarrhea, Berbere Woreda

## Abstract

**Background:**

A major cause of diarrheal illness mortality is a failure to seek immediate medical assistance. There is currently no evidence on the reasons that induce caregivers in Berbere Woreda to delayed seeking timely treatment for under-five children with diarrheal illnesses. As a result, the goal of this study was to identify determinants of delay in seeking timely treatment for childhood diarrheal diseases in Berbere Woreda, Bale Zone Oromia Region, South Eastern Ethiopia.

**Methods:**

An unmatched case–control study on 418 child caregivers was conducted from April to May 2021. Cases were 209 children and their caregivers who sought treatment after 24 h of the onset of diarrheal disease symptoms, and controls were 209 children and their mothers/caregivers who sought treatment within 24 h of the onset of diarrheal disease symptoms. Data were collected through interviews and chart reviews using consecutive sampling. A multivariable logistic regression analysis was carried out, with variables with a *P*-value of 0.05 considered statistically significant. The Hosmer–Lemshow goodness of fit test was used to validate the model, and the variance inflation factor (VIF) was used to test for multi-collinearity.

**Results:**

In this study, we found that among 418 participants, determinants of delay in seeking timely treatment for childhood diarrheal diseases included mothers with more than two under-five children (AOR = 2.23, 95% CI 1.21–4.11), Divorce (AOR = 2.62, 95% CI 1.087–2.76), age of children < 24 months (AOR = 1.597, 95%,CI (1.008–2.531), and preference for a government health facility for treatment (AOR = 2.56, 95% CI 1.51–4.34). Besides, the odds of mothers aged 25–34 years being two times more likely to delay seeking timely treatment for 5 children with diarrhea were 1.537 (0.560–4.213).

**Conclusions:**

Age of children, age of mothers, number of children, preference of health facilities, and marital status were factors influencing the failure to seek treatment within 24 h of recognizing diarrhea in children under the age of five.

## Background

The definition of diarrhea is three or more loose or watery stools in a 24-h period, or an increase in bowel frequency or fluidity that the mother of a child under the age of five considers abnormal. It is associated with a number of variables that lead to fluid loss, which increases the risk of infant mortality [1]. The top five global causes of illness and mortality in children under the age of five are all related to diarrhea and in 2017, 5.4 million children under the age of five lost their lives, with 78% of those deaths occurring in Africa and South Asia [2]. Furthermore, with 76 deaths per 1000 live births, Sub-Saharan Africa has the world's highest under-five mortality rate. The majority of African children die as a result of delayed medical attention [3].

According to a study done in Nairobi, Kenya, health-care seeking habits for diarrhea continue to offer substantial issues, with more than half (55%) of carers seeking improper health care and a high number of caregivers (35%) taking no action [4]. Similarly, according to a Tanzanian research, only 23.0% of children with acute diarrhea were treated in a health facility. Poor health-care seeking behavior promotes morbidity and mortality in children [5].

In Ethiopia, a significant percentage of children die as a result of delays in seeking medical attention. As a result, various studies have found that mothers/caregivers/of under-five children with diarrheal disorders seek less health treatment [6]. Furthermore, even when health care was sought, it was frequently delayed, resulting in morbidity and mortality among a large number of under-five children, as well as long-term complications [7]. There are a variety of reasons why people put off obtaining treatment for diarrheal illness. According to a Nigerian study, caregivers with formal education are 1.63 times more likely than those without formal education to get their child to a health institution on time [8].

Gender of the child, order of birth, and the number of children under the age of five were all factors influencing timely treatment seeking in Ethiopia. According to Demographic and Health Surveys from 2000 to 2016, a child's gender was significantly associated with rates of health-care seeking for childhood diarrhea illness [9]. In 2006, 51.8% of male children sought care; childbirth order 17% sought care for first birth order, 16.3% for second, 14.2% for third birth order, and total number of under-five births 51.3%, 12% sought care when under-five children were one and three and above [9]. Another study in Ethiopia found that reduced waiting times, 35.0% among patients and 44.5% among controls, were the main reasons for choosing a health institution [10]. A study conducted in Southwest Ethiopia found that households located within 10 km of a health center were three times as likely than those located more than 10 km away to seek health care. Rural families that experienced acute sickness were nine times more likely than those that felt chronic disease to seek health care [5].

In Ethiopia, health-care seeking has increased from 13% in 2000 to 22% in 2005, 32% in 2011, and 44% in 2016 [11]. However, both nationally and in the study area, research on the factors that cause mothers/caregivers of sick children to delay seeking timely health care is limited. Although several studies on the determinants of treatment seeking delay have been conducted in various parts of the world, none have been conducted in Berbere Woreda, Bale zone, Ethiopia. As a result, this study is required to determine the factors that influence delayed treatment seeking for childhood diarrheal disorders in public health facilities in Berbere Woreda, Bale Zone, South east Ethiopia.

## Methods and materials

### Study area and study subjects

From April to May of 2021, we conducted an unmatched case–control study in Berbere, Bale zone, Ethiopia. Consecutive sampling was used to recruit respondents for both cases and controls based on the time caregivers arrived at the health center after recognizing signs/symptoms of diarrheal diseases. Mothers/caregivers with under-five children diagnosed with diarrhea who arrived at the health center after 24 h of recognizing the onset of sign/symptoms were designated as cases [10, 12, 13], while mothers/caregivers with under-five children diagnosed with diarrhea who arrived at the health center before 24 h of recognizing the onset of sign/symptoms were designated as controls [10, 12, 13]. All mothers/caregivers paired with children with diarrheal disease who present to a respected health center within 24 h of the onset of signs/symptoms and give verbal consent; caregivers who have no medical problem (mental problem) that prevents them from providing accurate information are included in the study. Mothers/caregivers accompanied by children who arrive at health centers within 24 h of the onset of signs/symptoms, children with hearing disabilities, and readmitted children who were included in the previous visit were excluded.

The sample size was determined using the Epi Info Version 7 software and a sample size calculation formula. The sample size was calculated using the child's age in months as the main exposure variable because it yields a larger sample size, with 74.1% and 60.1% exposure among cases and controls, respectively [12]. 80% of power, 95% confidence level, AOR of 1.9, 1–1 case-to-control ratio, it yields sample size of 380 (Table 1), considering 10% non-response rate, the final total sample size was 418.

**Table 1:** Sample size determination

Factors	AOR	Proportion of controls	Ratio	Baseline *n* sample	Reference (source)
Age	1.9	60.1	1:1	380	Degefa et al. [12] Study in central Ethiopia, 2018
HF preference	2.9	82.3	1:1	326	Degefa et al. [12] Study in central Ethiopia, 2018
Caregivers literacy	2.4	17.1	1:1	254	Degefa et al. [12] Study in central Ethiopia, 2018
Having counseling	4.8	19.6	1:1	72	Degefa et al. [12] Study in central Ethiopia, 2018

### Data collection procedure

Following the completion of the diagnosis, data were collected using a translated and pretested structured questionnaire administered by an interviewer to assess predisposing, enabling, disease-related factors, and promptness of treatment seeking for childhood diarrhea. A structured questionnaire was used by data collectors to collect information on predisposing, enabling, disease-related factors, and promptness of treatment seeking in children with diarrhea. We used the patient history card to record vital signs and diarrhea complications. Cases and controls were determined using the chief complaint on the patient's card. After being diagnosed with diarrhea, cases and controls were recruited from hospitals and health centers/clinics. Cases and controls were recruited from hospital and health centers/clinics after they came to the facilities and were diagnosed with diarrhea. The information was gathered under the supervision of trained supervisors. Madda Walabu University's Institutional Review Board, Public Health and Environment, provided ethical approval. A letter was written to Oromia Health Beauru for approval, then to the Bale Zone Health Office, and finally to the Berbere Woreda Health Office. Berbere Woreda Health Office wrote a permission letter to Health Centers, and actual study participants provided informed verbal consent.

### Data quality control

We used a pretested structured and standardized questionnaire. A language expert translated the questionnaire into the local language (Afan Oromo) after it was created in English. For 2 days, we trained data collectors and supervisors. We conducted a pre-test on 5% of the total sample size before collecting data. Based on the results of the pre-testing, necessary revisions were made to the timing, skipping pattern, and coherence.

### Data processing and analysis

Data were collected and entered the data into Epidata Version 3.1 and exported to SPSS version 20 for further analysis. Bivariate logistic regression analysis was performed to examine the association between the independent variables and delay of health-care seeking, and odds ratios with 95% confidence intervals were calculated for all variables entered into the bivariate. The multivariable logistic regression analysis was employed to determine the independent predictors of delay in timely diarrhea treatment seeking of mothers/caregivers of under five children with diarrheal diseases at a *P*-value < 0.05 and 95% confidence interval of odds ratio.

All variables found significantly associated with health-care seeking at *P*-value < 0.25 in the bivariate analysis were entered into the multivariable model, and adjusted odds ratios with the 95% confidence intervals corresponding to variables included in a model were calculated. Model goodness of fit was checked using the Hosmer–Lemshow test of goodness of fit (× 2 = 14.047, *P*-value = 0.694, omnibus likelihood test (0.000) with 81.2% model accuracy. Multi-collinearity test was carried out and no problem of multi-collinearity test found as VIF < 2.5 and tolerance test > 0.25. Finally, variable with *P*-value < 0.05 in multivariable analysis was considered significant determinants.

### Operational explanation

*Timely treatment seeking*: Care that was obtained from medical facilities within 24 h of learning that young children under five had diarrhea [10, 12, 13].

*Treatment delay*: Care sought from health facilities after 24 h of recognizing the existence of diarrhea in children under the age of five [10, 12, 13].

*Care seeking*: Any treatment sought from a designated governmental or nongovernmental health facility for a child suffering from diarrheal illnesses [10, 12, 13].

## Results

### Socio demographic characteristics

In this study, 418 mothers/caregivers (209 cases and 209 controls) were interviewed making a 100% response rate. The majority (72.2%) of the cases and (61.2) of controls were children ≤ 24 months. The mean ± SD child age of cases was 18.37 (± 7.23), and that of control was 19.86 (± 8.33) months. More than half of mother/caregivers of case (74.2%) were with female children while 51.7% of mothers in controls were with male children (Table 2).

**Table 2:** Sociodemographic characteristics of under-five children in the Berbere, Bale, Southeastern Ethiopia, 2021

Variable	Patient category	
	Controls (*n* = 209) number (%)	Cases (*n* = 209) number (%)
*Age of child in months*		
≤ 24	151 (72.2)	128 (61.2)
> 24	58 (27.8)	81 (38.8)
*Sex of child*		
Male	101 (48.3)	54 (25.8)
Female	108 (51.7)	155 (74.2)
*Age of mother/caregiver*		
15–20	8 (3.8)	18 (8.6)
21–24	23 (11)	69 (33)
25–34	163 (78)	106 (50.7)
≥ 35	15 (7.2)	16 (7.7)
*Ethnicity*		
Oromo	199 (95.2)	200 (95.7)
Amhara	7 (3.3)	7 (3.3)
Somali	3 (1.4)	2 (1)
*Marital status*		
Married	189 (90.4)	178 (85.2)
Widowed	10 (4.8)	6 (2.9)
*Religion*		
Orthodox	39 (18.7)	39 (18.7)
Protestant	14 (6.7)	21 (10)
Muslim	156 (74.6)	149 (71.3)
*Place residence*		
Rural	129 (61.7)	152 (72.7)
Urban	80 (38.3)	57 (27.3)
*No of* < *5 children*		
≤ 2	197 (94.7)	168 (80.4)
> 2	12 (5.3)	41 (19.6)
*Birth order*		
≤ 2	61 (29.2)	61 (29.2)
3–4	119 (56.9)	110 (52.6)
≥ 5	7 (3.3)	38 (18.2)
*Education status of mother*		
No formal education	64 (30.6)	103 (49.3)
Primary education	132 (63.2)	95 (45.5)
Secondary and above	13 (6.2)	11 (5.3)

About 78% of the mother’s/caregivers controls were between ages of 25 and 34 and 50.7% of cases mother/caregiver falls in this age range. 95.7% of cases and 95.2% of controls were Oromo ethnicity, and 149 (71.3%) of cases were Muslims. More than three fourths of cases 178 (85.2%) and controls 189 (90.4%) were married. Almost half 103 (49.3%) cases has no education while more than half (63.2%) controls mother/caregivers attended primary education (Table 2).

Almost two-third 155 (74.2%) of cases and 160 (76.6%) controls were housewives while 18 (8.6%) cases. The majority (67%) of controls and (56%) cases preferred government health facilities. Distance and laboratory service 87 (71.9%) among case and 98 (67.6%) in controls were the major reason of preferring health center (Table 3).

**Table 3:** Determinants factors of delay in treatment seeking for under-five diarrheal disease in Berbere, Bale, Southeastern Ethiopia, 2021

Variable	Patient category	Patient category
	Controls (*n* = 209) number (%)	Cases (*n* = 209) number (%)
*Average monthly income (USD)*		
≤ 30	32 (15.3)	55 (26.3)
31–49.9	98 (46.9)	89 (42.6)
≥ 50	79 (37.8)	65 (31.1)
*Occupation of mother/caregiver*		
House wife	160 (76.6)	155 (74.2)
Farmer	18 (8.6)	22 (10.5)
Merchant	5 (2.4)	18 (8.6)
Government worker	26 (12.4)	14 (6.7)
*Distance from HF*		
< 5 km	131 (62.7)	99 (47.4)
5–10 km	61 (29.2)	80 (38.3)
≥ 10 km	17 (8.1)	30 (14.4)
*Cost of treatment*		
Very expensive	6 (2.9)	10 (4.8)
Expensive	15 (7.2)	36 (17.2)
Fair	179 (85.6)	150 (71.8)
Low	9 (4.3)	13 (6.2)
*Waiting time*		
Very short	6 (2.9)	8 (3.8)
Short	76 (36.4)	68 (32.5)
Fair	110 (52.6)	110 (52.6)
Long	17 (8.1)	23 (11)
*Previous similar illness*		
Yes	66 (31.6)	46 (22.7)
No	143 (68.4)	157 (77.3)
*Service available*		
Medicine	10 (18.9)	16 (19.8)
Laboratory service	19 (35.8)	29 (35.8)
HE/counseling	7 (13.2)	6 (7.4)
Clinical examination	17 (32.1)	30 (37)
*Response for illness/when do decide to seek Rx*		
As soon as seen any sign/symptoms	96 (45.9)	32 (15.3)
When see blood in stool	6 (2.9)	8 (3.8)
When frequent watery diarrhea	87 (41.6)	134 (64.1)
Child get weak & vomit every thing	20 (9.6)	35 (16.7)
*How is general service of this H/C*		
Good	166 (79.4)	151 (72.2)
Very good	22 (10.5)	9 (4.30)
Fair	21 (10)	49 (23.40)

### Need/disease related factors

Most of the mothers/caregivers take their child to the health facility 134 (64.1%) of cases, and 987 (41.6%) of control responded for frequent watery diarrhea while 35 (16.7%) of cases and 20 (9.6%) of control respond when child get weak or vomit everything. The majority (97.6%) cases and (98.6) of control respondents perceived diarrhea can hurt child (Table 3).

### Determinants of delay in timely treatment seeking

Bi-variable and multivariable logistic regression presented with crude and adjusted odds ratio, 95% CI for the variables that predict timely treatment seeking of mothers/caregivers of under-five children with diarrhea (Table 4). Multivariable logistic regression shows that sex and age of child, preference of health facility, taking children to health facility, birth order, number of under-five children, having counseling service on previous visit, mothers of younger children with diarrheal diseases were determinants to delay in timely seek treatment.

**Table 4:** Determinants of delay in treatment seeking for under-five children diarrhea in Berbere Woreda Bale, Southeastern, Ethiopia, 2021

Variable	Control	Cases	COR (95%CI)	AOR (95%CI)
*Sex*				
Female	108 (51.7)	155 (74.2)	2.684 (1.778–4.052)	2.947 (1.874–4.635)
Male	101 (48.3)	54 (25.8)	1	1
*Child age category*				
≤ 24 months	151 (72.2)	128 (61.2)	0.654 (1.092–2.486)	0.607 (1.421–1.017)
> 24	58 (27.8)	81 (38.8)	1	1
*Age of mother/caregiver*				
15–20	8 (3.8)	18 (8.6)	1	1
21–24	23 (11)	69 (33)	3.000 (0.041–3.179)	1.363 (0.015–3.200)
25–34	15 (7.2)	106 (50.7)	3.140 (0.124–0.701)	1.537 (0.560–4.213)
≥ 35	163 (78)	16 (7.7)	0.440 (0.159–1.411)	0.528 (0.129–2.158)
*Marital status*				
Married	189 (90.4)	178 (85.2)	1	1
Widowed	10 (4.8)	6 (2.9)	0.637 (0.178–1.584)	0.112 (0.033–3.204)
Divorced	10 (4.8)	25 (12)	2.654 (1.240–5.684)	2.628 (1.087–2.767)
*No of* < *5 children*				
≤ 2	197 (94.7)	168 (80.4)	1	1
> 2	12 (5.3)	41 (19.6)	4.006 (2.039–7.872)	3.695 (1.712–7.974)
Mother education status				
No formal education	64 (30.6)	103 (49.3)	1.900 (0.866–0.2.114)	2.617 (1.641–4.172)
Primary education	132 (63.2)	95 (45.5)	0.850 (0.036–0.224)	0.656 (0.050–0.109)
Secondary and above	13 (6.2)	11 (5.3)	1	1
*Cost of treatment*				
Very expensive	6 (2.9)	10 (4.8)	0.867 (0.231–3.250)	0.950 (0.129–2.903)
Expensive	15 (7.2)	36 (17.2)	0.503 (0.179–1.416)	0.512 (0.012–2.240)
Fair	179 (85.6)	150 (71.8)	1.440 (0.443–4.676)	0.011 (0.329–3.115)
Low	9 (4.3)	13 (6.2)	1	1
Waiting time				
Short	76 (36.4)	68 (32.5)	1	1
Long	17 (8.1)	23 (11)	0.750 (0.252–2.233)	0.784 (0.215–2.340)
*Service available*				
Medicine	10 (18.9)	16 (19.8)	0.954 (0.358–2.540)	1.002 (0.006–5.234)
Laboratory service	19 (35.8)	29 (35.8)	0.536 (0.139–2.058)	0.626 (0.650–3.302)
HE/counseling	7 (13.2)	6 (7.4)	0.480 (0.410–2.966)	0.328 (0.226–0.477)
No both laboratory and clinical service	17 (32.1)	30 (37)	1	1
Preference of HF				
Government health facility	159 (76.1)	113 (54.1)	2.701 (1.744–4.027)	2.563 (1.512–4.345)
Private clinic	50 (23.9)	96 (45.9)	1	1

Mothers those have greater than two under-five children were four times more likely to delay in timely treatment seeking than counterparts (AOR = 3.695, 95%, CI 1.712–7.974). In addition, mothers aged 25–34 years were two times more likely to delay seeking timely treatment for 5 children with diarrhea 1.537 (0.560–4.213). Mothers/caregivers who divorced their marriage were nearly three times more likely than mothers/caregivers who were married to delay seeking treatment for under-five children with diarrhea (AOR = 2.628, 95%, CI 1.087–2.767). Mothers/caregivers who preferred a government health facility for the treatment of their children with diarrhea were three times more likely to delay seeking treatment than those who preferred private health facilities (AOR = 2.563, 95%, CI (1.512–4.345). Moreover, mothers/caregivers with female children were almost three times more likely to delay for timely treatment seeking (AOR = 2.947, 95%, CI (1.874–4.635). Mothers/caregivers/ with ≤ 24 months’ children were more likely to delay for treatment than odds of those with > 24 months’ children (AOR = 1.597, 95%, CI (1.008–2.531) (Table 4).

## Discussion

In this study, treatment seeking for 5-year-olds with diarrheal disease was predicted by the child’s sex and age, mother and caregiver education, preference for health facilities, number of children under five, marital status of the mother and caregivers, maternal age, and counseling about the significance of visiting a health facility within and after 24-h of symptom onset. Mothers/caregivers with female children were three times more likely than those with male children to delay seeking therapy. This discovery is comparable to one seen in Nepal, where the gender of the kid influences treatment seeking [4, 14, 15]. On the contrary, this finding differed from studies in Nigeria that reported sex had no association with mothers/caregivers’ treatment seeking for under-five children [16]. The difference in seeking treatment for male and female children may be due to cultural influence and gender inequality that systematically disadvantages females in the community, which may influence a mother/ caregiver to prioritize a male child.

Mothers/caregivers with younger children (24 months) were 61% more likely to put off getting therapy than those with older children (24 months). The findings were congruent with those of a study conducted in central Ethiopia, which revealed that mothers/caregivers with children under the age of 24 months were twice as likely as mothers/caregivers with older children to delay accessing health-care assistance [17]. However, this finding is different from EDHS 2011 report [9]. This variation may be due to mothers/caregivers associating diarrhea with the eruption of milk teeth, while others believe it is due to frequent breast feeding and prefer to reduce breast feeding rather than seek treatment.

The maternal age group (25–34) was more likely to put off obtaining treatment for childhood diarrhea. This conclusion was comparable to one discovered in Ethiopia's Woliso district, where women aged (26–34) were more likely to delay obtaining treatment for childhood diarrhea [12]. However, this differs from a study conducted in Ethiopia in 2015 and Kenya in 2012, which discovered that mothers/caregivers of children sought treatment more frequently when they were in their twenties or early thirties [18]. The difference could be due to older caregivers having repeated episodes of diarrhea that distract them from the complications of diarrhea and expecting spontaneous recovery.

Children of mothers/caregivers with more than two children under the age of five had four times the odds of seeking treatment later than children of mothers/caregivers with two or fewer children under the age of five. In this finding similar to the findings of earlier studies, family size may influence mothers'/caregivers' health-care seeking behavior for pediatric illnesses [19]. This could be because mothers/caregivers with large family sizes pay less attention to sick children due to a heavy workload and limited resources.

Mothers’/caregivers’ educational status was also a determinant factor, with mothers who did not have formal education nearly three times more likely to delay seeking treatment than their counterparts. This is consistent with findings from studies conducted in central Ethiopia [20]. However, this is in contrary with a study conducted in Niger [8]. This variation may be due to the fact that mothers/caregivers who have at least completed primary school are thought to have a better opportunity to learn health information than those who have not completed primary school. Furthermore, mothers/caregivers who have not received formal education may lack basic knowledge about the consequences of delaying treatment. Mothers/caregivers who were counseled about the importance of seeking treatment for children's diarrhea at a health facility on time were 67% less likely to delay than their counterparts. This is consistent with the findings of an Ethiopian study, which found that mothers and caregivers benefit from prior information when visiting health facilities for childhood diarrhea [10]. This finding, however, contradicts a study conducted in Rwanda, which found that mothers/caregivers of under-five children with diarrheal illness who received their first consultation at a public health institution were more likely to be delayed [4]. This could be because caregivers of under-five children who are informed about complications and the benefits of early treatment care may pay attention to even minor symptoms and bring their child as soon as they notice them.

The preference of health facility for treatment of children with diarrheal diseases was approximately three times more likely to delay in those who preferred a government health center over those who preferred a private clinic. This is comparable to a study conducted in low-income countries, which discovered a high rate of consultation at private providers due to factors such as convenience, prompt care, and more courteous service, which influence treatment seeking in government health facilities [14]. However, this differs from a study conducted in Ethiopia, which found that government health facilities are more likely to be sought for the treatment of children with diarrhea because the costs are relatively low for mothers/caregivers [12]. This could be due to the fact that most clients who prefer health centers may wait to connect with their own upcoming follow-up or integrate with other activities such as marketing.

The cost of treatment, waiting time, and perceptions of the overall service of a health facility were not major predictors. Other Ethiopian studies have revealed that household income and health-care facility expenses are determinants of health-care seeking among mothers/caregivers with children under the age of five [20]. The difference could be due to the ability to pay for health-care facilities, which may be less variable among these respondents, as the majority report that the costs of treatment are affordable.

### Limitation of the study

This study has own strength being case–control study by nature of study design and using large sample size of heterogeneous population. Still this study has own limitations that it was restricted to governmental health facilities and did not include private clinics. The case and control group classifications were based on the duration of onset of symptoms (chief complaints) recorded on the patient card, which may have resulted in misclassification bias. Other limitation using unmatched case control, this find gain less efficiency due to nature of study where difficult totally to omit confiding effect. Many independent variables, such as health facility preference, previous visit counseling, and child age, rely on mothers’/caregivers’ responses, which may lead to recall bias. It only considers those who meet the inclusion criteria and evaluates a small number of determinants. This study selects cases and controls using a consecutive sampling technique, which may limit the study's generalizability.

## Conclusions

The age of the children, the age of the mothers/caregivers, the preference of health facilities, the first response to diarrhea, and a history of previous counseling about the importance of seeking treatment within 24 h of recognizing diarrhea were discovered to be determinants of the delay in seeking treatment within 24 h of recognizing diarrhea in under-five children sin this study. As a result, preventive care programs should prioritize age, health facility preference, and getting children with diarrhea to a health facility as soon as possible. To improve the timely treatment seeking of under-five children with diarrheal diseases, it is critical to raise awareness among mothers/caregivers about the importance of timely treatment seeking.

## Data Availability

All data generated or analyzed for this study are available from the corresponding authors upon reasonable request.
